# Alpha-l-Locked Nucleic Acid-Modified Antisense Oligonucleotides Induce Efficient Splice Modulation In Vitro

**DOI:** 10.3390/ijms21072434

**Published:** 2020-03-31

**Authors:** Prithi Raguraman, Tao Wang, Lixia Ma, Per Trolle Jørgensen, Jesper Wengel, Rakesh N. Veedu

**Affiliations:** 1 Centre for Molecular Medicine and Innovative Therapeutics, Murdoch University, Perth 6150 Australia; Prithi.Raguraman@murdoch.edu.au (P.R.); Tao.Wang@murdoch.edu.au (T.W.); 2 Perron Institute for Neurological and translational Science, Perth 6005, Australia; 3 School of Statistics, Henan University of Economics and Law, Zhengzhou 450001, China; realmlx@163.com; 4 Nucleic Acid Center, Department of Physics and Chemistry and Pharmacy, University of Southern Denmark, M 5230 Odense, Denmark; ptj@sdu.dk (P.T.J.); jwe@sdu.dk (J.W.)

**Keywords:** α-l-LNA, locked nucleic acids, antisense oligonucleotides, DMD

## Abstract

Alpha-l-Locked nucleic acid (α-l-LNA) is a stereoisomeric analogue of locked nucleic acid (LNA), which possesses excellent biophysical properties and also exhibits high target binding affinity to complementary oligonucleotide sequences and resistance to nuclease degradations. Therefore, α-l-LNA nucleotides could be utilised to develop stable antisense oligonucleotides (AO), which can be truncated without compromising the integrity and efficacy of the AO. In this study, we explored the potential of α-l-LNA nucleotides-modified antisense oligonucleotides to modulate splicing by inducing *Dmd* exon-23 skipping in *mdx* mouse myoblasts in vitro. For this purpose, we have synthesised and systematically evaluated the efficacy of α-l-LNA-modified 2′-O-methyl phosphorothioate (2′-OMePS) AOs of three different sizes including 20mer, 18mer and 16mer AOs in parallel to fully-modified 2′-OMePS control AOs. Our results demonstrated that the 18mer and 16mer truncated AO variants showed slightly better exon-skipping efficacy when compared with the fully-23 modified 2′-OMePS control AOs, in addition to showing low cytotoxicity. As there was no previous report on using α-l-LNA-modified AOs in splice modulation, we firmly believe that this initial study could be beneficial to further explore and expand the scope of α-l-LNA-modified AO therapeutic molecules.

## 1. Introduction

**Figure 1:**
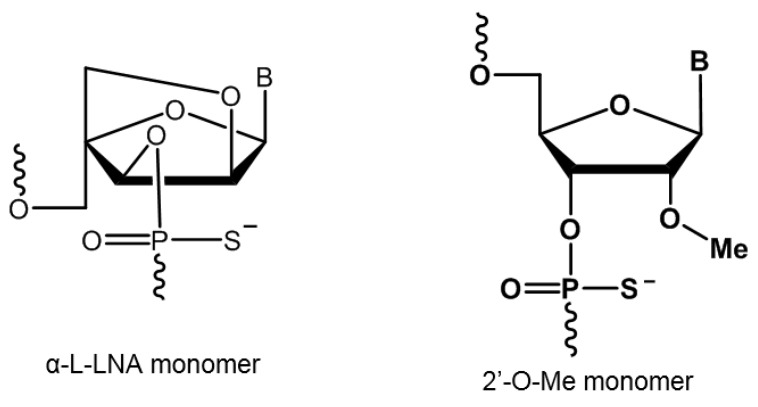
Structural representation of the phosphorothioate nucleic acid analogues used in this study.

Alpha-l-locked nucleic acid (α-l-LNA) is a stereoisomeric analogue of locked nucleic acid (LNA) with the inverted stereochemistry at C2′, C3′ and C4′ positions (Figure 1). Like LNAs, α-l-LNA also exhibits high binding affinity to complementary RNA and DNA oligonucleotides when incorporated into oligonucleotides [1,2]. Apart from this, α-l-LNA nucleotides also displayed a high degree of resistance to nucleases in addition to showing decreased cytotoxicity [3]. The favourable biophysical properties of α-l-LNA have led to several studies towards evaluating their potential, including for therapeutic applications [4,5,6,7,8].

Duchenne muscular dystrophy (DMD) is a serious muscle-wasting disorder, caused by lack of dystrophin protein, which is essential for the normal functioning of the muscle cells, including muscle contraction and movement. Dystrophin acts as an anchor, connecting the cytoskeleton of the muscle fibre and the extracellular matrix. Lack of dystrophin protein usually occurs due to mutations in one or more exons or deletion of exons of the dystrophin gene [9]. Antisense oligonucleotide (AO)-mediated splice modulation has been well explored to reinstate the reading frame in order to produce the partially functional dystrophin protein [10,11,12]. One such drug, Exondys 51 (Sarepta Therapeutics) composed of phosphorodiamidate morpholino oligomer (PMO) chemistry, was conditionally approved in 2016 by the US Food and Drug Administration (FDA) for clinical use [13]. Although the PMO drug is approved for DMD, the production cost of PMOs is extremely high, and importantly, it is not compatible with standard phosphoramidite chemistry. This problem could be addressed by developing shorter phosphorothioate AOs, which could help reduce the cost of the AOs. It is worth adding here that a parallel trial conducted for another AO candidate Drisapersen composed of 2′-OMePS chemistry (Figure 1) (BioMarin Pharmaceutical) was rejected by the FDA [14]. Herein, we report the systematic evaluation of α-l-LNA modified 2′-OMePS AOs and their efficiency to induce exon-skipping in *mdx* mouse myotubes in vitro.

## 2. Results

First, we synthesised 20mer α-l-LNA-modified 2′-OMePS AO (NAC 9078) containing three α-l-LNA nucleotides at positions 15, 18 and 20 and their corresponding fully-modified 2′-OMePS control AO (20mer, 3642). The AOs were then systematically truncated by removing one and two nucleotides respectively from the 5′ and 3′ ends to produce 18mer AOs (NAC 9079 with three α-l-LNA modification at positions 13, 15 and 18, and NAC 9080 with five α-l-LNA nucleotides at positions 2, 7, 10, 14 and 17) and a 16mer AO (NAC 9081 with three modifications at positions 12, 14 and 16) as well as their 2′-OMePS controls (18mer, 4036; 16mer, 4039). The binding affinity of the AOs against the complementary RNA target was examined by performing a thermal stability analysis (Table 1). The *Dmd* exon-23 skipping efficiency of the AOs was then analysed in vitro using the *H-2K^b^*-tsA58 *(H2K) mdx* mouse myotubes. Briefly, the cells were plated on a 24-well plate and allowed to differentiate into myotubes over 24 h. These cells were then transfected with the AOs using Lipofectin, a cationic lipid-based delivery agent, at 2.5, 5, 12.5, 25, 50 and 100 nM concentrations. The treated cells were collected after 24 h of incubation, and subsequently the RNA was isolated followed by RT-PCR analysis. The PCR products were later analysed by 2% agarose gel electrophoresis. The gel images were quantified by performing a densitometry analysis using the ImageJ software.

### 2.1. Evaluation of α-l-LNA AOs to Induce Exon-23 Skipping

**Figure 2:**
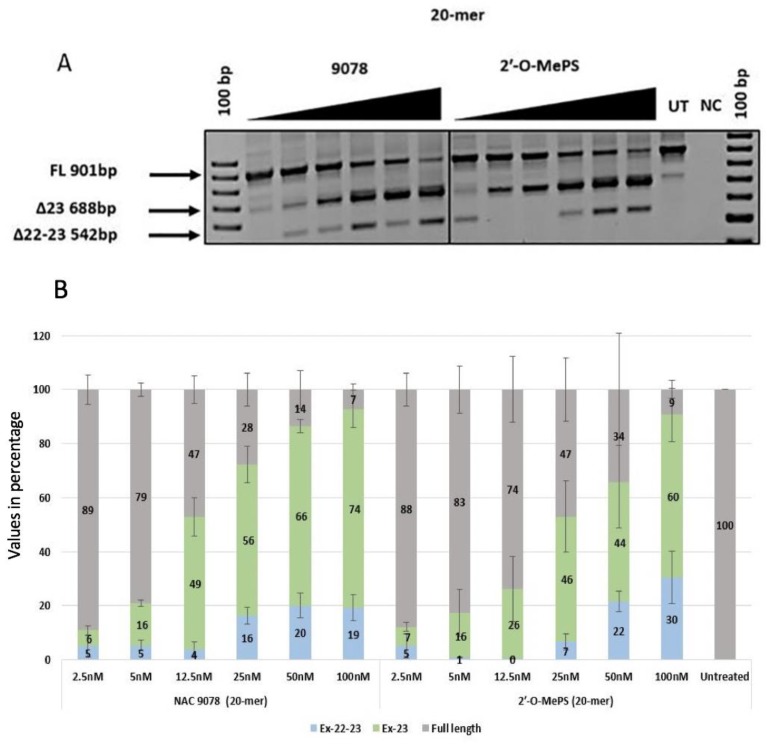
(**A**) RT-PCR analysis of exon-23 skipping induced by antisense oligonucleotides (AO) NAC 9078 and the corresponding 2′-OMePS control AO in *H2K mdx* mouse myotubes (gel image A corresponds to one of the three gels used for data in (**B**)); (**B**) densitometry analysis of exon-23 skipping induced by AO NAC 9078 and the corresponding 2′-OMePS control AO (triplicates) in *H2K mdx* mouse myotubes. Concentration range 2.5–100 nM. Grey, full-length product exon 20–26; green, exon-23 skipped product; blue, dual exon 22/23 skipped product; UT, untreated; NC, negative control (Error bars represent the standard deviation of mean).

The exon-23 skipping efficiency of α-l-LNA modified 2′-OMePS AOs was evaluated in parallel to the corresponding 2′-OMePS control unmodified AOs of similar lengths. The 20mer NAC 9078 containing three α-l-LNA nucleotides with a melting temperature (*T*_m_) of 65.2 °C (Table 1) showed efficient exon-23 skipping (Figure 2). The exon skipping efficiency was similar or even better when directly compared with the corresponding 2′-OMePS AOs (Figure 2A). Highest exon skipping was observed at 50 nM and 100 nM with 66% and 74% of the 688 bp skipped transcript product (Figure 2B).

**Figure 3:**
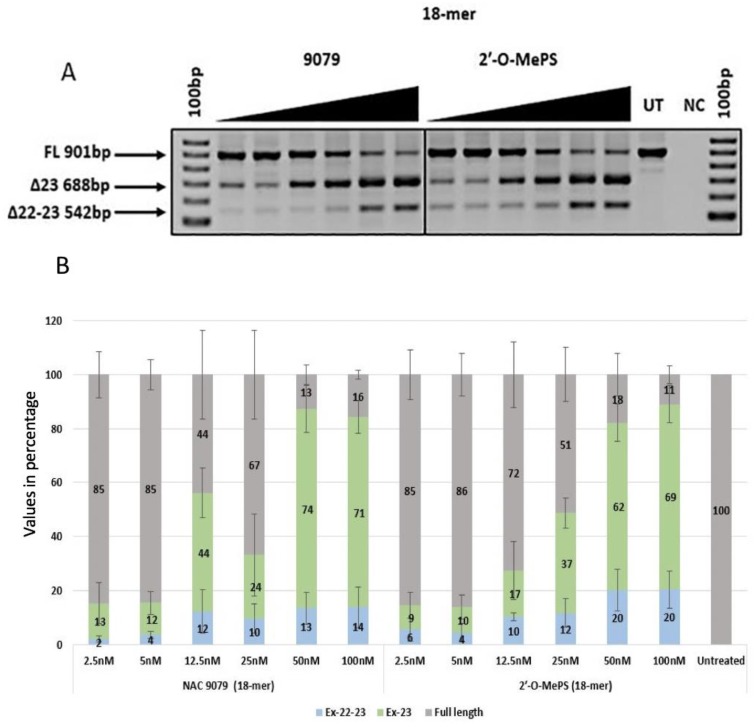
(**A**) RT-PCR analysis of exon-23 skipping induced by AO NAC 9079 and the corresponding 2′-OMePS control AO in *H2K mdx* mouse myotubes (gel image A corresponds to one of the three gels used for data in (**B**); (**B**) densitometry analysis of exon-23 skipping induced by AO NAC 9079 and the corresponding 2′-OMePS control AO (triplicates) in *H2K mdx* mouse myotubes. Concentration range 2.5–100 nM. Grey, full-length product exon 20–26; green, exon-23 skipped product; blue, dual exon 22/23 skipped product; UT, untreated; NC, negative control (Error bars represent the standard deviation of mean).

**Figure 4:**
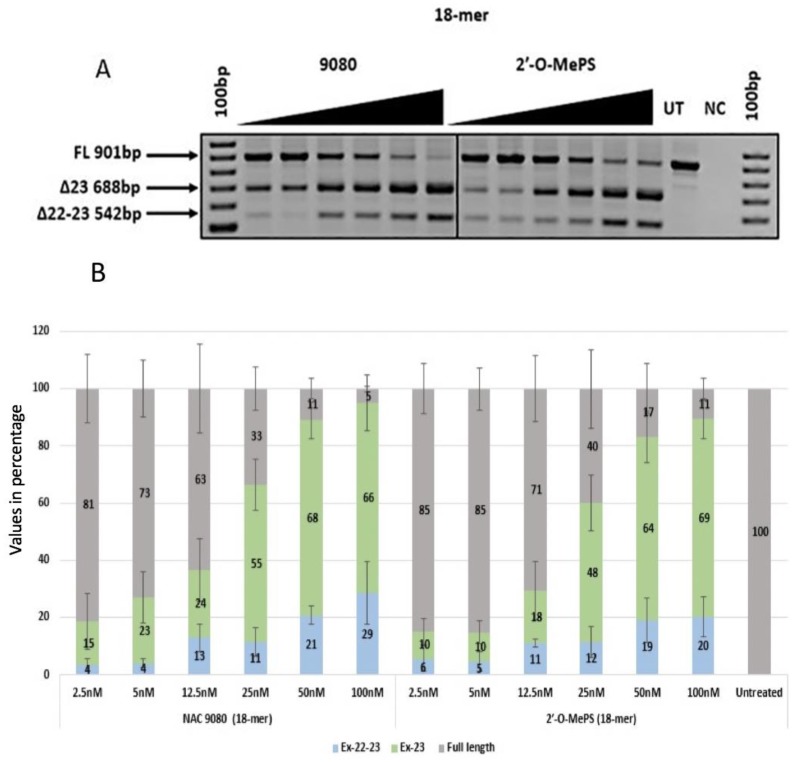
(**A**) RT-PCR analysis of exon-23 skipping induced by AO NAC 9080 and the corresponding 2′-OMePS control AO in *H2K mdx* mouse myotubes (gel image A corresponds to one of the three gels used for data in (**B**)); (**B**) densitometry analysis of exon-23 skipping induced by AO NAC 9080 and the corresponding 2′-OMePS control AO (triplicates) in *H2K mdx* mouse myotubes. Concentration range 2.5–100 nM. Grey, full-length product exon 20–26; green, exon-23 skipped product; blue, dual exon 22/23 skipped product; UT, untreated; NC, negative control (Error bars represent the standard deviation of mean).

Similarly, both the 18mer AOs NAC 9079 with three α-l-LNA nucleotides and NAC 9080 with five α-l-LNA nucleotides induced better exon skipping when compared with the corresponding 2′-OMePS AOs. Both the α-l-LNA modified AOs had higher *T*_m_ of 64.2 °C and 68.8 °C when compared with the control 2′-OMePS unmodified AOs with a *T*_m_ of 57.7 °C (Table 1) and induced efficient exon-23 skipping at all tested concentrations (Figure 3A and Figure 4A). Notably, α-l-LNA modified AOs NAC 9079 (74% and 71%) and NAC 9080 (68% and 66%) induced better exon-23 skipping at 50 nm and 100 nM respectively (Figure 4B) than the control 18mer 2′-OMePS AO (62% and 69% at 50 nM and 100 nM respectively, Figure 3B).

**Figure 5:**
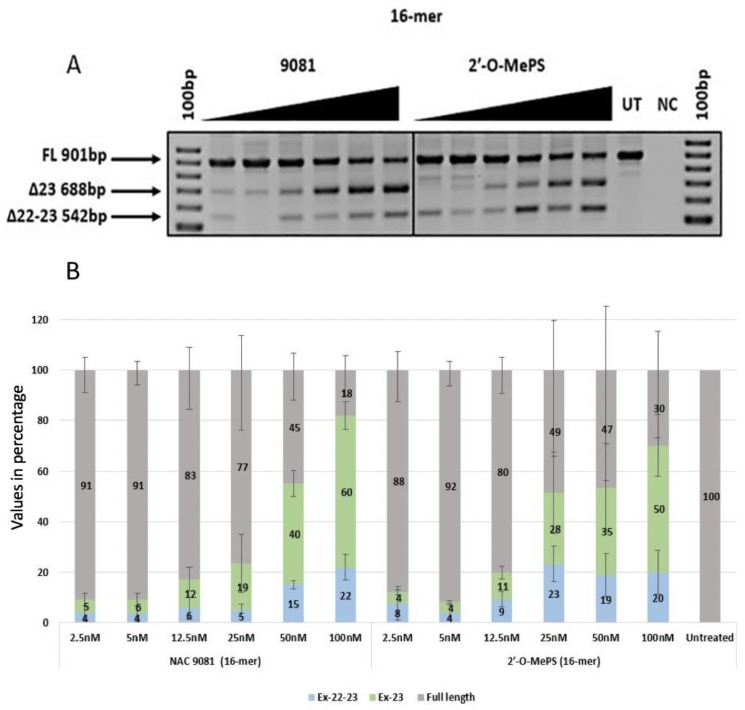
(**A**) RT-PCR analysis of exon-23 skipping induced by AO NAC 9080 and the corresponding 2′-OMePS control AO in *H2K mdx* mouse myotubes (gel image A corresponds to one of the three gels used for data in (**B**); (**B**) densitometry analysis of exon-23 skipping induced by AO NAC 9080 and the corresponding 2′-OMePS control AO (triplicates) in *H2K mdx* mouse myotubes. Concentration range 2.5–100 nM. Grey, full-length product exon 20–26; green, exon-23 skipped product; blue, dual exon 22/23 skipped product; UT, untreated; NC, negative control (Error bars represent the standard deviation of mean).

The 16mer AO NAC 9081 containing three α-l-LNA nucleotides exhibited a much higher *T*_m_ of 59.6 °C when compared to the control 16mer 2′-OMePS AO (*T*_m_: 53.1, Table 1). In this case, the α-l-LNA modified AO demonstrated to be better in inducing exon-23 skipping at all tested concentrations (2.5–100 nM) compared with the control 2′-OMePS AO (Figure 5A,B). Interestingly, NAC 9081 demonstrated highest exon-23 skipping efficacy at 50 nM (40%) and 100 nM (60%) concentrations, whereas, the corresponding 2′-OMePS control AO only yielded 35% and 50% respectively, demonstrating the impact of α-l-LNA nucleotides modifications (Figure 5B).

In addition to the exon-23 skipped transcript (688 bp), all the AOs also seemed to induce dual exon 22/23 skipping (542 bp), and the intensity of the dual (exon 22/23) skipped bands increased with the increasing concentrations. The undesired dual exon skipping could lead to a shorter dystrophin transcript. In general, the dual exon skipping yield was much lower when compared to the expected exon-23 deletion products (Figure 2B, Figure 3B, Figure 4B, Figure 5B and Table 2). The dual skipping induced by α-l-LNA modified AOs was either comparable or even lesser at certain concentrations when compared to the corresponding control 2′-OMePS AOs (Figure 2A, Figure 3A, Figure 4A and Figure 5A). The efficiency of exon skipping was also evaluated by an EC50 assay (Figure 7).

### 2.2. Evaluation of Cytotoxicity of AOs

**Figure 6:**
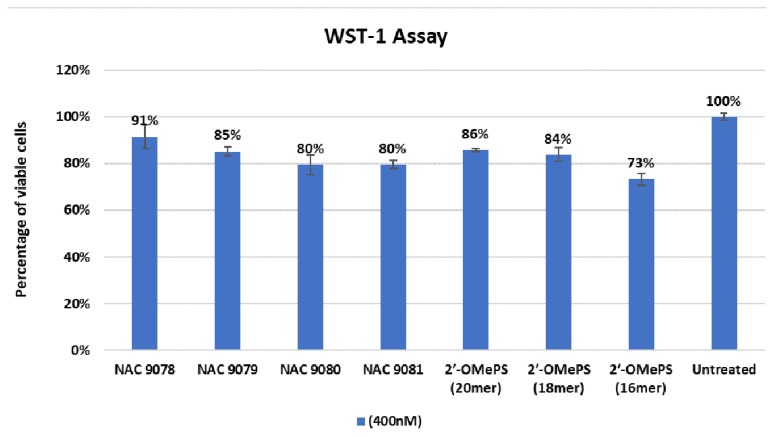
Percentage of viable cells observed after transfection quantified using a WST-1 assay (Error bars represent the standard deviation of mean).

A dye (WST-1)-based cell viability assay was performed to assess the cytotoxicity of the α-l-LNA modified AOs on the *H2K mdx* mouse myoblast. In parallel, the control 2′-OMePS AOs were also analysed for the cell toxicity. The AOs were all tested at the highest concentration of 400 nM. Results clearly showed that α-l-LNA modified AOs were relatively less cytotoxic compared with the corresponding control 2′-OMePS AO controls (NAC 9078, 91%; NAC 9079, 85%; NAC 9080, 80%; NAC 9081, 80%; controls: 20mer, 86%; 18mer, 84% and 16mer, 73%, as seen in Figure 6). To further analyse the potential cytotoxicity (IC50) of the AOs, MTT assay was performed. As shown in the supplementary Figure S1 (Supplementary Information), a similar trend was recorded for all tested AOs in *mdx* mouse myoblasts.

## 3. Discussion

Modulation of RNA splicing using synthetic antisense oligonucleotide has been established as a viable therapeutic strategy for tackling diseases [15]. This approach has been well studied in DMD system in vitro and in vivo over the last two decades. In 2016, an antisense-drug named Eteplirsen (Exondys 51) composed of PMO chemistry was conditionally approved by the US FDA for the treatment of DMD [13]. Another drug candidate entered in Phase 3 trials, Drisapersen (2′-OMePS chemistry), was rejected by the FDA due to the failure to meet the primary and secondary endpoints and also due to the increased renal and hepatotoxicity [14], which reinforces the requirement for alternative approaches to improve the efficacy of the 2′-OMePS chemistry. Towards this goal, various chemically-modified nucleic acid analogues have been explored previously [16,17,18,19,20,21,22,23]. In this study, we explored the scope of α-l-LNA analogues to induce exon-skipping. Fluiter et al. showed that α-l-LNA AOs had better efficacy to downregulate H-Ras for tumour inhibition in vitro. Furthermore, the AOs were also found to be nontoxic. Also, these α-l-LNA-modified AOs effectively inhibited tumour growth in vivo at minimal dosage of 0.5 mg/kg [1]. In addition, our lab previously reported that systematically truncated LNA-modified AOs demonstrated very high efficacy to induce exon-23 skipping in the DMD system in vitro [18]. In line with that study, it was also important to evaluate the potential of α-l-LNA being a stereoisomeric analogue of LNA in splice modulation.

In this study, we used α-l-LNA modified 2′-OMePS sequences, which were systematically truncated and evaluated for their efficacy to induce exon-23 skipping in *mdx* mouse myoblasts in vitro in parallel to the corresponding 2′-OMePS control AOs. For this purpose, we used previously reported 20mer AO (NAC 9078) designed to target mouse exon-23, which was modified by incorporating three α-l-LNA nucleotides [17]. The AOs were then truncated by removing one to two nucleotides from the 3′ and 5′ ends to obtain 18mer (NAC 9079) and 16mer (NAC 9081) truncated variants containing three α-l-LNA nucleotides. We also constructed an additional 18mer AO with five α-l-LNA nucleotide incorporations (NAC 9080). The modifications were placed towards the 3′-end of the sequences, in line with a previous report [17]. The results indicated that all the AOs were efficient in inducing exon-23 skipping in a dose-dependent manner at all tested concentrations (2.5 nM–100 nM). The 18mer AO NAC 9080 with five α-l-LNA modifications and NAC 9079 with three α-l-LNA modifications were found to be the best in inducing exon-23 skipping, which might reflect its targeting affinity (*T*_m_ of 68.8 °C and 64.2°C respectively). Although at higher concentrations the exon-23 skipping efficiency of NAC 9078, NAC 9080 and NAC 9079 were comparable, NAC 9080 still is the more appealing due to its shorter sequence length and higher number of modifications when compared to NAC 9078 and NAC 9079. It was also observed that at lower concentrations, the 18mer AOs NAC 9079 and NAC 9080 performed better than the 20mer NAC 9078. Notably, the 16mer NAC 9081 performed much better than the corresponding 2′-OMePS sequence but the exon skipping efficacy was not as high when compared with the other modified 20mer and 18mer AOs. The pattern of exon skipping observed in the AOs compared to their corresponding control 2′-OMePS AOs can be found in Table 2. In general, the α-l-LNA modified AOs induced better exon skipping when compared to the 2′-OMePS AOs, and also showed less cytotoxicity (Figure 6).

**Figure 7:**
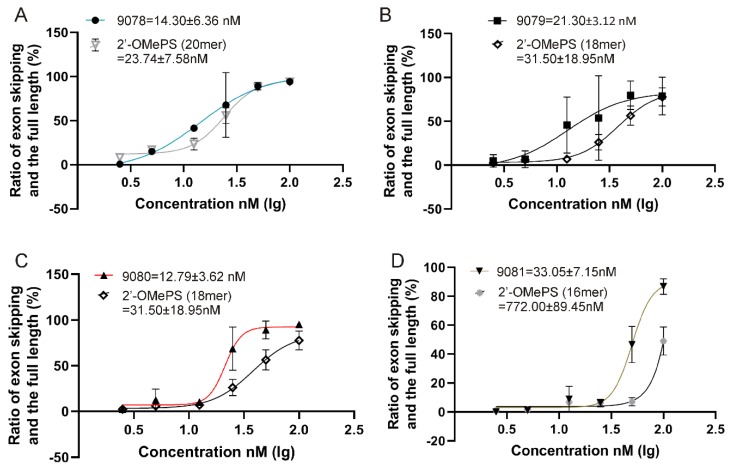
Evaluation of half maximal effective concentration (EC_50_) of the AOs vs. their corresponding 2′-O-methyl phosphorothioate (2′-OMePS) control AO (Error bars represent the standard deviation of mean). (**A**) NAC 9078 vs 2′-OMePS control AO (20mer); (**B**) NAC 9079 vs 2′-OMePS control AO (18mer); (**C**) NAC 9080 vs 2′-OMePS control AO (18mer); (**D**) NAC 9081 vs 2′-OMePS control AO (16mer).

To investigate the potential cell damage induced by the AOs NAC 9078, NAC 9079, NAC 9080 and NAC 9081 along with their respective controls, a cytotoxicity assay (IC_50_ analysis) was conducted using the *mdx* mouse myoblasts. As shown in Figure S1 (Supplementary Information), similar cytotoxicity was observed for all the AOs. With a concentration up to 1000 nM, a 50% inhibition rate (IC50) was not observed. These data indicated that all the compounds did not display prominent cytotoxicity in the dose ranges (from 2.5 nM to 100 nM) carried out in this study. To measure the exon skipping potency of the AOs, an EC50 assay was conducted using a series of concentrations from 2.5 nM to 100 nM. As shown in Figure 7 (Figure S2; Supplementary Information), NAC 9080, with an EC50 of 12.79 nM, recorded the highest exon skipping efficacy, followed by NAC 9078 (14.3 nM), NAC 9079 (21.3 nM) and NAC 9081 (33.05 nM). This outcome is in accordance with our AO transfection and exon-23 skipping results. In parallel, we performed a two-factor variance analysis (% exon-23 skipping vs. dose), compared with the controls. Although only NAC 9081 displayed statistical significance (*p* = 0.00807) when compared to its corresponding control, the other compounds showed a general trend of better skipping than controls (Figure S3; Supplementary Information). *H-2Kb-tsA58 mdx* cell line does not express functional dystrophin protein due to a non-sense mutation in exon 23 of *Dmd* gene transcript and limits the feasibility of protein assays including western-blot and immunofluorescence to show the effects of the AO-mediated expression of dystrophin protein in vitro after 24–48 h of transfection. Typically, in vivo study was performed for this purpose using higher doses and prolonged treatment periods. For example, in a comprehensive study by Fletcher et al., the AO was injected into *mdx* mice with doses from 2–10 μg over a 4-week period to visualise dystrophin protein expression [24].

In summary, we evaluated the potential of α-l-LNA modified 2′-OMePS AOs in splice modulation, and our results demonstrated that the modified AOs were slightly more efficient to induce exon-23 skipping compared with the corresponding control 2′-OMePS AOs. Our findings suggest that shorter α-l-LNA modified AOs are capable of increasing the efficiency of the 2′-OMePS AOs, and this analogue could also be used for developing splice-modulating antisense therapeutics in combination with other nucleotide chemistries.

## 4. Materials and Methods

### 4.1. Melting Temperature Analysis

All the oligonucleotides were prepared at 2 µM concentration in a buffer solution containing 0.01 mM EDTA and 10 mM NaCl. The buffer solution was adjusted to pH 7 using 10 mM sodium phosphate buffer. The prepared oligonucleotides were then mixed with an equal volume of the complementary RNA sequence at the same concentration. The mixture was then denatured for 10 min at 95 °C and gradually cooled down to room temperature. The mixture was then loaded on a quartz cuvette of 1 mm path length, and the melting point was observed using Shimadzu UV-1800 fitted with a temperature controller. The temperature was maintained over the range of 20–90 °C with a ramp rate of 1 °C min^−1^. The melting points of all the oligonucleotides (T_m_) were calculated by the first derivative. As we have used 2′-OMePS/RNA with higher stability as a control, the delta *T*_m_ increase observed with α-l-LNA was lower than previously reported.

### 4.2. Cell Culture and Transfection

*H-2K^b^*-tsA58 *(H2K) mdx* mouse myoblasts were grown as described before [25,26]. The primary mdx myoblast cells were trypsinised when they were about 60–80% confluent and about 25 × 10^3^ cells were seeded in 24-well plates. The plates were pre-treated with 50 µg ml^−1^ poly-d-lysine (Sigma Aldrich; Castle Hill, NSW, Australia) and 100 µg ml^−1^ Matrigel (In Vitro Technologies; Noble Park North, VIC, Australia). The cultures were then differentiated into myotubes in Dulbecco’s Modified Eagle Media (ThermoFisher Scientific; Riverstone, NSW, Australia) supplemented with 5% Horse serum by incubating in 5% CO_2_ at 37 °C for 24 h. The AOs were then complexed with lipofectin (ThermoFischer Scientific; Riverstone, NSW, Australia) at a ratio of 2:1 (lipofectin: AO) and was transfected at a final volume of 500 µl following the manufacturer’s protocols, except the solution was not removed after 3 h. The experiments were performed in three experimental repeats with replicas (Figure 2, Figure 3, Figure 4 and Figure 5).

### 4.3. RNA Extraction and RT-PCR

RNA was extracted from the transfected cells using the Isolate II RNA Mini Kit (Bioline; Eveleigh, NSW, Australia) following the manufacturer’s protocols. The dystrophin transcripts were analysed then by a nested-PCR across exons 20–26. The PCR products were separated on a 2% agarose gel in Tris-acetate-EDTA buffer, and the images were captured on a Fusion Fx gel documentation system (Vilber Lourmat, Marne-la Vallee, France). Image J software was used for the densitometry analyses.

### 4.4. Cell Viability and Cytotoxicity Assay

The cells were seeded at a density of 2 × 10^4^ cells per well and transfected with AOs at 400 nM, as mentioned before. After 24 h, the viability of the cells was analysed using a colorimetric assay (WST-1, Sigma Aldrich; Castle Hill, NSW, Australia). The WST-1 and optimum solution were added in the ratio 1:10 (*v/v*) in each well and incubated at 37 °C for 2 h, 5% CO_2._ Absorbance was measured at 450 nm with a microplate reader (FLUOstar Omega, BMG Labtech, Ortenberg, Germany).

MTT assay was conducted as previously reported [27]. Briefly, cells (3 × 10^3^ cells/well) in 200 µl of culture mediums were seeded in 96-well plates and incubated for 24 h. After that, the culture medium was replaced by medium containing Lipofectin and indicated oligos at indicated concentrations. After 24 h incubation, 5 mg/mL MTT reagent (Sigma Aldrich; Castle Hill, NSW, Australia) in 1 × PBS −20 µl/well (ThermoFisher Scientific; Riverstone, NSW, Australia) was added into the plates and incubated for 3 h. After incubation, the medium was aspirated and dimethyl sulfoxide −150 µl/well (Sigma Aldrich; Castle Hill, NSW, Australia) was added to stop the reaction. The absorbance was quantified by a FLUOstar Omega multi-detection microplate reader (BMG Labtech, Ortenberg, Germany) at 570 nm wavelength. The cell viability was calculated by comparing the luminescent signal of treatment group to the signal obtained with untreated cells (setting as 100% viability). Each value represents the mean standard deviation from duplicates.

### 4.5. Evaluation of Efficiency of Exon Skipping

*H-2Kb*-tsA58 *(H2K) mdx* mouse myoblasts were seeded in a 24-well plate pre-treated with 50 µg ml^−1^ poly-D-lysine (Sigma Aldrich; Castle Hill, NSW, Australia) and 100 µg ml^−1^ Matrigel (In Vitro Technologies; Noble Park North, VIC, Australia) 24 h before transfection. Next, the cells were transfected with the AOs NAC 9078, NAC 9079, NAC 9080 and NAC 9081 using Lipofectin (ThermoFischer Scientific; Riverstone, NSW, Australia) transfection reagent according to the manufacturer’s protocol at a series of concentrations including 100 nM, 50 nM, 25 nM, 12.5 nM, 5 nM and 2.5 nM. Twenty-four hours after transfection, the cells were collected for RNA extraction using Isolate II RNA Mini Kit (Bioline; Eveleigh, NSW, Australia) as per the manufacturer’s protocol. The transcripts were amplified by a nested-PCR across exons 20–26. The products were then separated on a 2% agarose gel in Tris-acetate-EDTA buffer, stained with Gel Red (Vazyme Biotech; Nanjing, China) and visualised with the Fusion Fx gel documentation system (Vilber Lourmat, Marne-la-Vallée, France). Densitometry analysis was performed by the ImageJ software. EC50 was calculated using different treatment concentrations and corresponding ratio of exon skipping and the full length via Graphpad Prism 8 (program: log vs. response—Find EC50). The comparison between the efficacy of individual AO compounds and their corresponding controls were analysed by the R program using the ggplot 2 Package (MathSoft, Cambridge, MA, USA).

## Figures and Tables

**Table 1:** Sequences used in this study.

AO Name	Sequence (5′-3′ direction)	*T*_m_ (°C)
NAC 9078 (20mer)	GGC CAA ACC UCG GCαT UAαC CαT	65.2
NAC 9079 (18mer)	GCC AAA CCU CGG αCUαT ACαC	64.2
NAC 9080 (18mer)	GαCC AAA αCCU αCGG CαTU AαCC	68.8
NAC 9081 (16mer)	CCA AAC CUC GGαC UαTA αC	59.6
2′-O-MePS (20mer)	GGCCAAACCUCGGCUUACCU	60.8
2′-O-MePS (18mer)	GCCAAACCUCGGCUUACC	57.7
2′-O-MePS (16mer)	CCAAACCUCGGCUUAC	53.1

Complementary synthetic RNA used in this study: 5′-r(AG GUA AGC CGA GGU UUG GCC)-3′. The α-l-LNA modified nucleotides are represented in red underlined letters, and U,C,A,G are 2′-O-methyl-RNA nucleotides.

**Table 2:** Summary of the percentage of exon 23 skipping and dual exon 22/23 skipping induced by the AOs. The mean of three repeats are represented in the table. Standard deviation is mentioned in brackets.

AO Name	Percentage of Exon 23 Skipping (%)						Percentage of Dual Exon 22/23 Skipping (%)					
	2.5 nM	5 nM	12.5 nM	25 nM	50 nM	100 nM	2.5 nM	5 nM	12.5 nM	25 nM	50 nM	100 nM
NAC 9078	6 (2.93)	16 (2.23)	49 (12.41)	56 (11.68)	66 (4.21)	74 (11.92)	5 (6.95)	5 (3.36)	4 (4.51)	16 (5.33)	20 (8.13)	19 (8.50)
NAC9079	13 (13.23)	12 (7.51)	44 (15.76)	24 (26.30)	74 (15.27)	71 (10.53)	2 (2.01)	4 (2.27)	12 (13.79)	10 (9.26)	13 (10.30)	14 (12.94)
NAC 9080	15 (17.13)	23 (15.42)	24 (18.90)	55 (15.52)	68 (11.23)	66 (17.12)	4 (3.62)	4 (2.53)	13 (8.43)	11 (8.79)	21 (5.55)	29 (18.86)
NAC 9081	5 (4.08)	6 (4.35)	12 (8.10)	19 (19.71)	40 (9.13)	60 (9.29)	4 (5.02)	4 (5.26)	6 (7.64)	5 (4.37)	15 (2.95)	22 (8.75)
2′-O-MePS (20mer)	7 (3.10)	16 (15.06)	26 (20.88)	46 (22.81)	44 (29.25)	60 (17.13)	5 (7.68)	1 (0.34)	1 (0.18)	7 (5.06)	22 (6.65)	30 (16.92)
2′-O-MePS (18mer)	10 (7.96)	10 (6.83)	18 (17.62)	48 (16.86)	64 (15.38)	69 (12.22)	6 (7.39)	5 (5.99)	11 (2.41)	12 (9.07)	19 (13.45)	20 (11.89)
2′-O-MePS (16mer)	4 (1.79)	4 (1.04)	11 (4.32)	28 (27.75)	35 (29.98)	50 (21.53)	8 (11.34)	4 (5.10)	9 (5.06)	23 (12.06)	19 (14.58)	20 (14.81)

## Supplementary Materials

The following are available online at https://www.mdpi.com/1422-0067/21/7/2434/s1, Figure S1: Evaluation of IC50 of the indicated compounds using mouse myoblasts, Figure S2: Evaluation of EC50 of the indicated compounds using mouse myoblasts, Figure S3: Graphs obtained after evaluation exon skipping efficiency of the control Vs the AOs using a two-factor variance analysis.

## Abbreviations

AO	Antisense oligonucleotide
α-l-LNA	Alpha-L-locked nucleic acid
DMD	Duchenne muscular dystrophy
