# Supplementary material for: Alpha-l-Locked Nucleic Acid-Modified Antisense Oligonucleotides Induce Efficient Splice Modulation In Vitro
Source: Int J Mol Sci. 2020 Mar 31;21(7):2434. doi: 10.3390/ijms21072434 (PMC7177859; doi:10.3390/ijms21072434)
Supplement: Supplementary file 1 [file ijms-21-02434-s001.pdf]

Supplementary Information

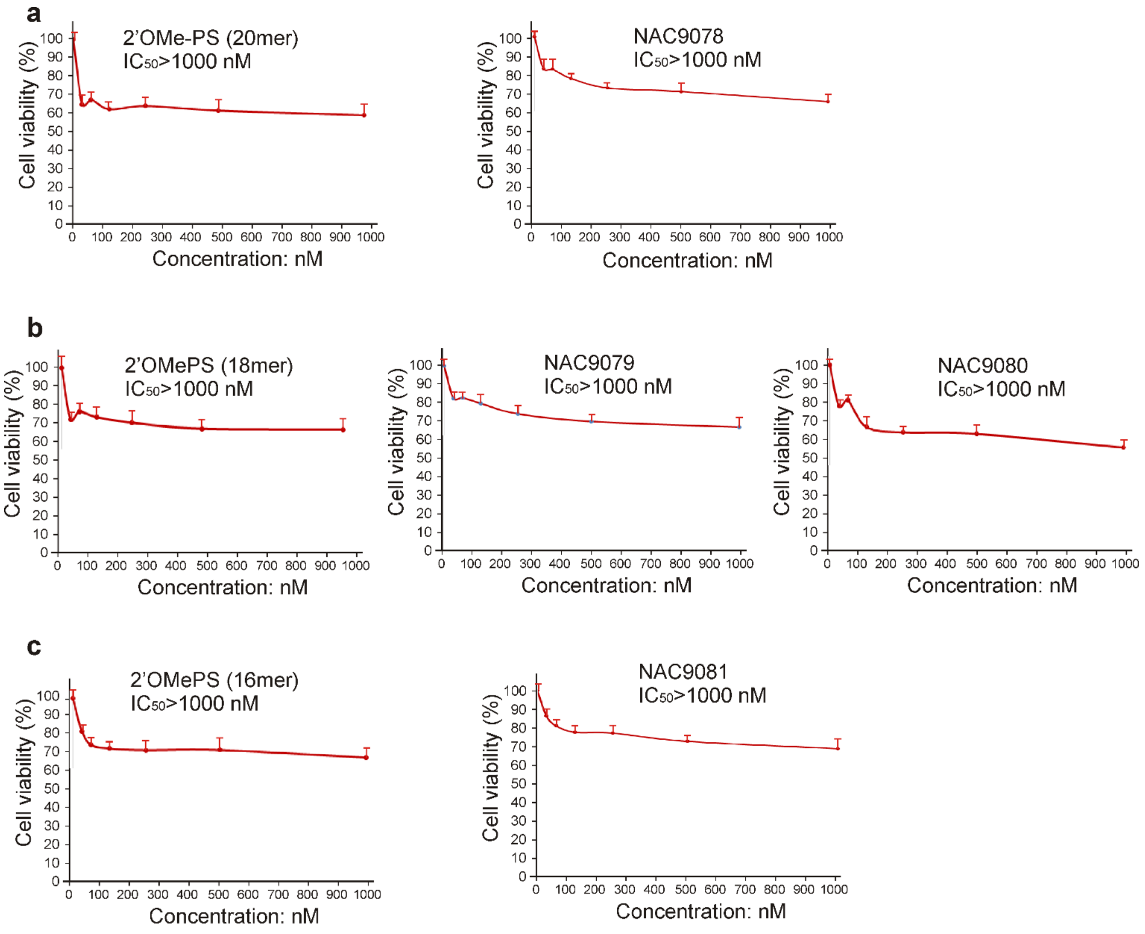

Figure S1. Evaluation of IC50 of the indicated compounds using mouse myoblasts.

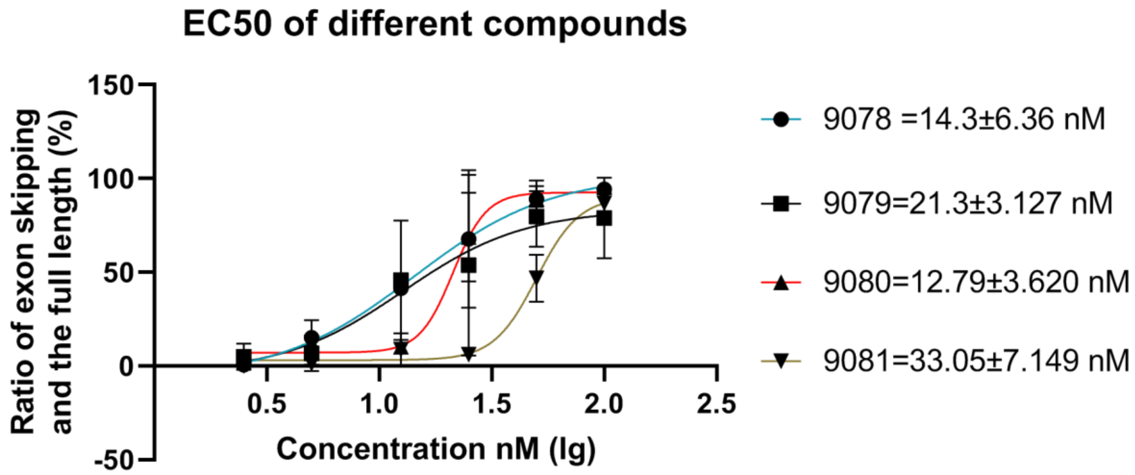

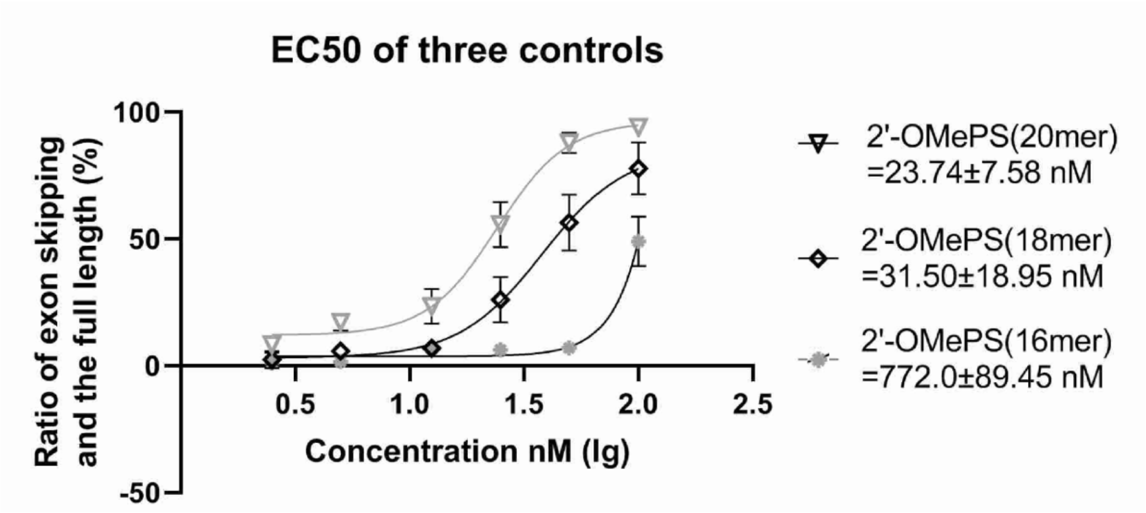

Figure S2. Evaluation of EC50 of the indicated compounds using mouse myoblasts.

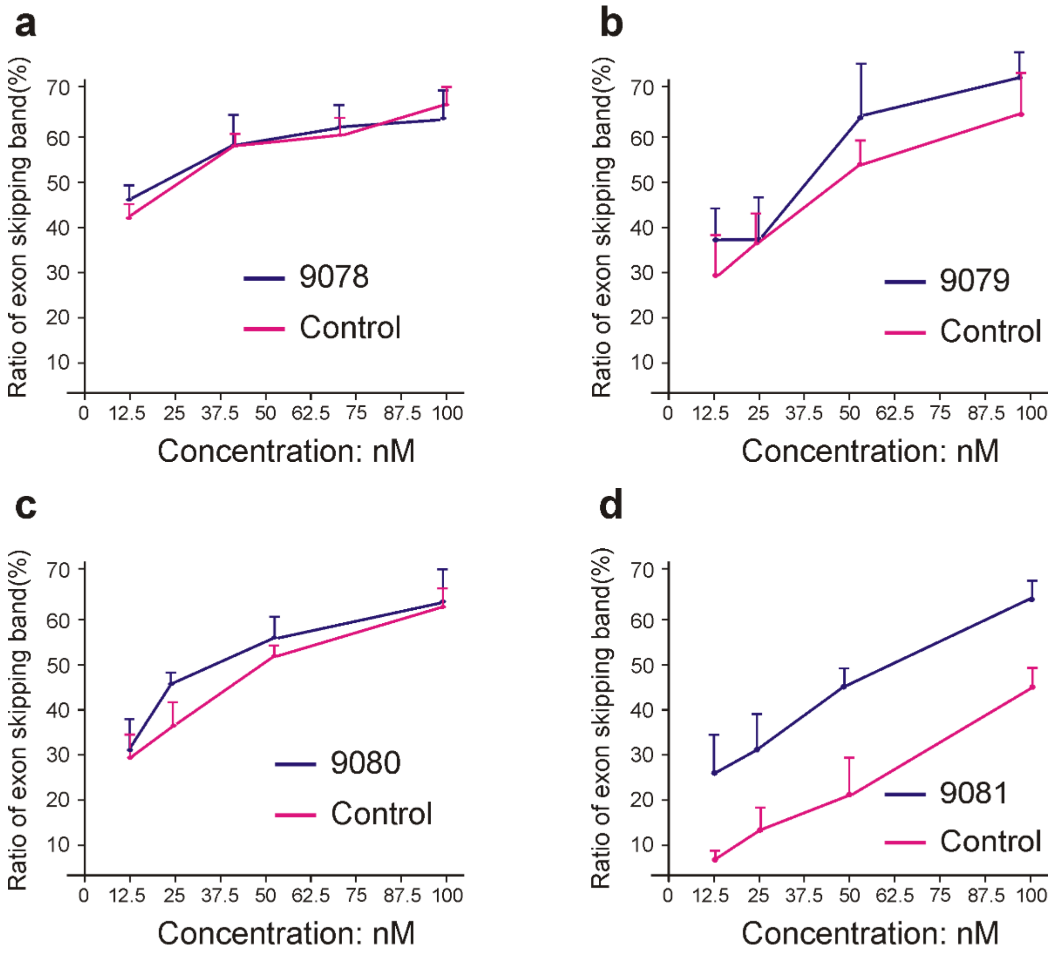

Figure S3. Graphs obtained after evaluation exon skipping efficiency of the control Vs the AOs using a two-factor variance analysis.
